# VDA, a Method of Choosing a Better Algorithm with Fewer Validations

**DOI:** 10.1371/journal.pone.0026074

**Published:** 2011-10-12

**Authors:** Francesco Strino, Fabio Parisi, Yuval Kluger

**Affiliations:** 1 Department of Pathology, Yale University School of Medicine, New Haven, Connecticut, United States of America; 2 New York University Center for Health Informatics and Bioinformatics, New York, New York, United States of America; Kings College, London, United Kingdom

## Abstract

The multitude of bioinformatics algorithms designed for performing a particular computational task presents end-users with the problem of selecting the most appropriate computational tool for analyzing their biological data. The choice of the best available method is often based on expensive experimental validation of the results. We propose an approach to design validation sets for method comparison and performance assessment that are effective in terms of cost and discrimination power.

Validation Discriminant Analysis (VDA) is a method for designing a minimal validation dataset to allow reliable comparisons between the performances of different algorithms. Implementation of our VDA approach achieves this reduction by selecting predictions that maximize the minimum Hamming distance between algorithmic predictions in the validation set. We show that VDA can be used to correctly rank algorithms according to their performances. These results are further supported by simulations and by realistic algorithmic comparisons *in silico*.

VDA is a novel, cost-efficient method for minimizing the number of validation experiments necessary for reliable performance estimation and fair comparison between algorithms.

Our VDA software is available at http://sourceforge.net/projects/klugerlab/files/VDA/

## Introduction

The analysis of complex biological systems requires a large investment of both funding and time [1]–[5]. The wealth of data being collected from biological systems often surpasses human capabilities to find patterns without using computerized analysis pipelines. Bioinformatics is the driving force in the design and exploration of novel, efficient and reliable algorithms to recover signals and patterns in biological experiments [6]–[16]. However, discordance between the predictions of available algorithms is a widespread phenomenon, resulting in difficulty selecting the most accurate algorithm. Independent experimental validation of algorithmic predictions can, in principle, provide adequate information to choose the best available method for a study. However, end-users (e.g. experimentalists) typically focus on using a single existing algorithm and assess its performance by performing a limited number of validation experiments [17].

On the other hand, comparative studies have relied on simulated data or on pre-existing validation datasets [10], [11], [14]–[16], [18]. Pre-existing validation datasets obtained from earlier studies are typically prepared to assess and fine-tune the performance of a single algorithm [17]. Since algorithms are often fine-tuned in a recursive process to attain the best performance on a specific set of validation data, these datasets may be inappropriate for unbiased comparison of algorithmic performance.

As the number of available algorithms increase, a new design for validation sets becomes necessary to achieve fair comparisons, and, most importantly, aid researchers in the selection of the best analysis tool available. In principle, one could test all predictions from all algorithms and estimate the performance of each algorithm. However, in most applications and particularly in genomics, the large number of validation experiments required for such assessment makes this approach unfeasible. The main limitation is the cost of the validation experiments, and, in some cases, the time needed to perform them; while running a different algorithm on the same dataset can be done quickly at virtually no cost, adding several new validation experiments can certainly be costly.

This problem is common to many fields of science besides genomics. It is particularly useful for event detection in one dimensional signal analysis. For example, the time course of one dimensional ECG or EEG signal can be divided into time segments, denoted as negatives, where the signal is regular (no event) and time segments, denoted as positives, where it is irregular (event). Similarly, in genomics experiments such as ChIP-seq analysis the density of the reads along the genome constitute a one-dimensional signal. In this scenario the genome coordinates can be segmented and divided into two sets: the set of segments for which a protein-DNA binding take place (event), and the set of segments for which there is no binding (no event). With the advent of high-throughput approaches, it is compelling to have a procedure for the design of a minimal set of validation experiments that enable comparison of several algorithms in a cost-effective fashion. These validation experiments should constitute an independent validation set to help choose between existing algorithms rather than fine-tune a novel method. We term this procedure validation discriminant analysis (VDA) and we propose an algorithmic framework intended to provide a very small set of experiments to discriminate different algorithms with high confidence and assess their performance. Our studies indicate that our proposed method for VDA is superior in convergence and discriminatory power to validation sets constructed by random selection.

VDA is a general approach, not limited to any field of science, and is most beneficial when one analytical method has to be chosen from a pool of available existing algorithms to make predictions where independent experimental validation is expensive. To the best of our knowledge, our algorithm for VDA is the only tool for designing cost efficient sets of validation experiments capable of discriminating between several algorithms and of estimating their accuracy.

## Results

### Selecting predictions to rank algorithmic performances

The main purpose of VDA is to judiciously select a compact set of instances for independent experimental validation in order to reliably rank the predictive power (performance) of a group of algorithms. In the present study we focus on ranking algorithms that are designed to predict the presence or absence of a certain phenomenon (See section A of Appendix S1).

Often algorithms can be fine-tuned by changing some parameters at run-time. For the present study, we will assume a black-box approach, where the methods used have optimal default parameters and thus fixed sensitivity and fixed specificity. Using a black-box approach reduces the Receiver-Operator Curve (ROC), a frequently used indicator of performance, to one point, the operative point. The corresponding Area Under the Receiver Operator Characteristic Curve (AUCROC) at the operative point can be computed using the common trapezoidal interpolation and the result is equal to the balanced accuracy, which is the average of sensitivity and specificity. The AUCROC at the operative point is hereby denoted by 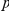. The function 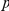 is a measure of algorithmic performance and it can be calculated for the 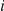-th algorithm as follows:
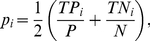
(1)where 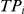 and 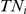 are the number of true positives and true negatives of the 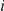-th algorithm, corresponding to the total number of correct predictions that an event occurred, or did not occur respectively; 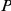
and 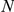 are the number of positives and negatives as determined by experimental validation.

We rank the performance of algorithms by detecting differences between their AUCROCs. These differences can be estimated by performing validation experiments for a subset of instances and compare the readout of these validations with the predictions of the algorithms in this subset of instances. Therefore, the best validation set contains the instances that have a high probability of resulting in significant absolute differences between many pairs of AUCROCs, where the absolute difference between a pair of AUCROCs is denoted by 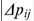 and defined, for two algorithms 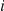 and 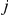, as
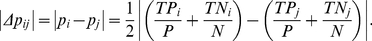
(2)


Importantly, 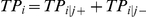, where 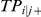 is the number of true positives that are predicted correctly by both algorithms, and 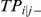 is the number of true positive predictions for the 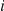-th algorithm that are false negatives for the 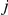-th algorithm. The absolute difference 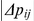, therefore, can be rewritten as
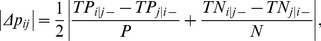
(3)where 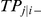
and 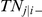
are the true positives and true negatives for the 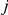-th algorithm for experiments in which the 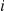-th and 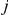-th algorithm have opposite predictions. Equation 3 recapitulates the fundamental point that the difference in performance between two algorithms can only be determined by experimental validation of discordant predictions.

Due to the operative cost of validation, validation outcomes, namely 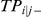, 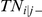, 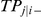 and 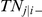, are not known *a priori*. On the other hand, their sum 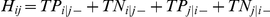 is known and it is equal to the number of discordant predictions between the 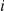-th and 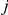-th algorithm. We define 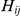 as the Hamming distance between the predictions of the two algorithms. The set of predictions whose experimental validation results in a non-zero absolute difference of performances 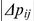, is a subset of all predictions, including those not selected for validation, that contribute to the Hamming distance 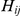. It is important to remark that 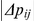 is not a measure of algorithmic performance, which should be instead computed as described below in Equation 5.

Given a set of *M* algorithms, we define the set *U'* of detectable predictions as the collection of all instances where at least one algorithm inferred the occurrence of an event. It is important to note that the set *U'* depends on the chosen algorithms and that the set *U* of all possible occurrences of events, regardless of the algorithm chosen, could be too large to explore or even unknown. In this regard, *U'* is a subset of *U*. Importantly, we define the set *D* of all instances for which at least two algorithms give discordant predictions, with 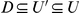. Differences in performance can only be detected by validating instances from the subset *D*.

Random sampling from the set *D* can generally result in a validation set whose experimental validation discriminates algorithmic performances. However, alternative selection strategies may enable us to achieve the same discrimination power as random sampling with fewer experimental validations. We call the identification of compact yet algorithmically discriminative validation sets VDA (for Validation Discriminant Analysis).

### Ranking algorithm using the VDA set

The main purpose of a VDA validation set is to enable ranking of algorithms based on their performance. In the present study we used the AUCROC at the operative point as a measure of performance as described in Equation 1. In Equation 5 below, we discuss an important modification of Equation 1 to enable robust estimation of AUCROC scores when validation experiments cannot be performed on all instances and sampling is used to design validation sets.

### Greedy Algorithm for VDA

Greedy VDA (GVDA) is an iterative procedure that utilizes the predictions made by *M* algorithms to sort instances according to their potential to maximize discrimination between the *M* algorithms. For a validation study comprising of *X* validation experiments, we select the first *X* sorted instances. Validation of these *X* selected instances enables quick convergence to the true ranking of algorithmic performances. Stability in ranking can be fine-tuned by selecting a larger number of instances for experimental validations. GVDA iteratively selects the prediction that maximizes the minimum Hamming distance 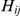 in the validation set between all pairs 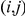 of algorithms. Among the available choices, GVDA selects the instance that will also recursively increase the other Hamming distances between the other pairs of algorithms in the validation set, starting from the smallest 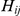. These requirements can be elegantly satisfied by selecting at the 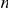-th iteration the instance 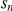 such that
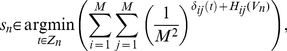
(4)where 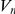 is the set of instances that have already been included in the VDA set, 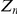 is the difference set between the set of discordant predictions *D* and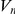, 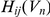 is the Hamming distance between the 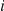-th and the 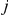-th algorithm in the set 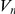, and 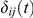 is an indicator variable that takes the value 1 if the 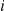-th and 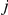-th algorithm have a discordant prediction for the instance 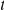, and 0 otherwise. If several choices are available with the same score, GVDA selects one at random. To reduce the search space, we group all available instances into clusters. All the instances within a given cluster share the same fingerprint: an M-dimensional binary vector whose 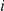-th element indicates whether the prediction made by the 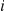-th algorithm for each of these instances is classified as positive (1) or negative (0). A step-by-step example of GVDA is presented in section B of Appendix S1. The complexity of GVDA is quadratic in the number of algorithms and linear (at each iteration) in the number of fingerprints. The subsets based on GVDA sorting are designed to be better than or equal to randomly sampled subsets of equal size in terms of discriminatory power. However, for a chosen subset size *X*, the possibility of a more discriminatory subset of instances than the one identified using GVDA cannot be entirely excluded.

### Exhaustive Algorithm for VDA

Exhaustive VDA (EVDA) is an algorithm for VDA designed to identify the best validation set of a given size. This algorithm is particularly useful in laboratory settings where the number of possible validations is subject to budgetary limitations. EVDA is similar to GVDA in design, although it implements a dynamic programming exhaustive recursive search with branching and memoization [19]. EVDA takes the desired minimum Hamming distance 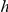 and a set of possible experiments *D* as input and returns the smallest subset 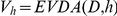 that satisfy that condition. The set 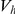 is then sorted by iteratively calculating the subsets 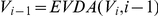
and concatenating the obtained subsets in the following order 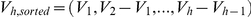. If 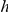 is not specified, EVDA uses the minimum Hamming distance between two algorithms. EVDA has quadratic complexity in the number of algorithms, and exponential complexity in the number of instances, making it unsuitable for large datasets, such as the one used in the present study. It should be noted that the degree of improvement from GVDA to EVDA is rather limited. For these reasons, in the present study we only use the GVDA algorithm.

### VDA set and random sampling enable estimation of the same AUCROC

Validation Discriminant Analysis is a non-random sampling procedure to select instances for experimental validation. Due to the built-in selection bias, a correction is needed to enable accurate estimation of algorithmic performances. Notably, given a partition set of the data, namely a group of non-overlapping and non-empty sets of instances, such that the union of these sets corresponds to the entire data, the AUCROC in Equation 1 can be rewritten as the AUCROC of weighted averages of *TP* and *TN* over the partition set of the data (See section C of Appendix S1 for derivation and an illustrative example). For a given algorithm:
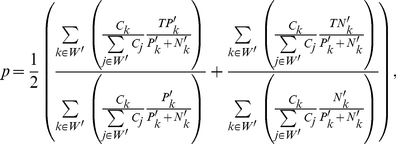
(5)where *W* is the partition set of *U*; 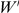 is the subset of *W* for which at least one validation experiment is available; 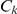
is the cardinality of the 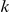-th partition of *U*, namely the number of instances in the partition; 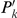
and 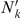 are the experimentally validated positives and negatives in the 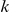-th partition; 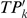 and 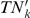 are the confirmed true positives and true negatives in the 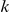-th partition of *U*. In the case of partial experimental validation, only a subset of instances from each partition is tested.

### VDA set enables correct AUCROC ranking with an equal or better rate compared to random sampling

To study the rate of convergence rate to the correct AUCROC estimation, we consider two groups of RDFA algorithms, RDFA(*a*) and RDFA(*b*) with 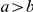, and we show that using VDA it is possible to discriminate the two groups of algorithms at a quicker rate than using random sampling. These two groups of algorithms can be represented as lines in the ROC plane parallel to the diagonal at distance 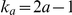 and 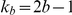, respectively. Without loss of generality, we can consider two specific RDFAs such that 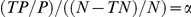 and, according to Equation 1, write the rate at which the two AUCROCs diverge as a function of the number of negatives in the validation set: 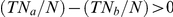.

From the definition of discordant predictions, the following inequalities are derived:
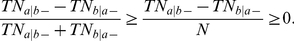
(6)


These inequalities indicate that on the set D, the two AUCROCs diverge faster (left term) than on the full set of all available predictions (middle term), corresponding to a standard agnostic random sampling approach. However, if the AllTrue and AllFalse algorithms are also included to guarantee correct AUCROC estimation, the rate for the difference 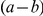 becomes
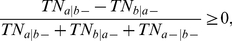
(7)where 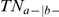 is the number of negatives that are wrongly predicted by both algorithms and that are selected by the VDA procedure in order to discriminate from the AllTrue and AllFalse algorithms. In general, the rate in Equation 7 may not be better than the middle term in Equation 6. However, we expect it to be better for VDA based on the following considerations. First, since 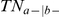 is selected to increase the Hamming distance between at least any two algorithms, the average rate of divergence between all algorithms in Equation 7 will always be superior to the average rate of divergence of the middle term in Equation 6. Second, in the case of large disparities in the sizes of partitions, random sampling will sample from each partition with probability proportional to its size, thus requiring a large number of validations to explore some configurations of predictions. A VDA approach will instead sample from the relevant partitions uniformly, regardless of their actual frequency, allowing quick and uniform exploration of the differences between algorithms.

### VDA exhibits superiority for data with largely imbalanced partitions

**Figure 1:**
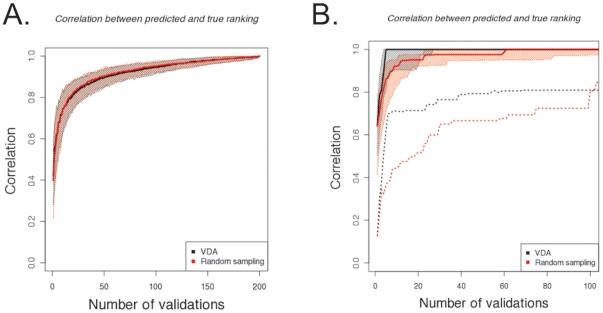
Comparison between VDA and random selection strategies in identifying validation experiments that enable correct ranking of algorithmic performances. The Kendall t-statistics between the inferred ranking and the true ranking of AUCROCs is used to assess the goodness of performance inference. The statistics from the VDA validation set are shown in black and the statistics from the randomly sampled validation set are shown in red. The medians of the t-statistics for each size of the validation set are shown as a bold line. The shaded regions correspond to the area between the first and the third quartiles. A. VDA and random selection of validation experiments have equivalent performances ranking 6 RDFA algorithms when the data can be partitioned into well-balanced subsets. B. VDA is superior to random sampling in correct ranking of performances of 6 RDFA algorithms when the data is not partitioned into well-balanced subsets. The trend of the fifth percentile (dotted lines) shows that VDA has a slower worst-case convergence to correct ranking than random sampling.

To test the validity of the VDA approach in ranking and discriminating algorithmic performances, we want to confirm that VDA is at least equal to random sampling in terms of cost, and we therefore test the conditions in which a VDA approach can offer a valuable improvement. We first verify that VDA and random sampling have the same performance on a dataset with well-balanced partitions, such that the number of predictions in each partition is roughly the same. Specifically, we simulate 200 sets of predictions of 6 RDFA algorithms (Random Detectors with Fixed AUCROC, see Methods for definition) with increasing AUCROC performances between 0 and 1 and with varying ratios between positives and negatives. It is easy to verify that when the performances of the 6 RDFA algorithms are equally spaced in the interval 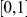, the partitions obtained by fingerprinting are well balanced. We evaluate how the Kendall correlation of the predicted AUCROC improves as a function of the number of validated predictions and find that there is no significant difference between the VDA and the random sampling strategy (Figure 1A). However, in datasets where the partitions are not balanced the VDA approach shows remarkable improvement in estimating correct ranking of performances as demonstrated by selecting RDFA target performances at random between 0 and 1 (Figure 1B).

### Comparing algorithms with identical performances using VDA

**Figure 2:**
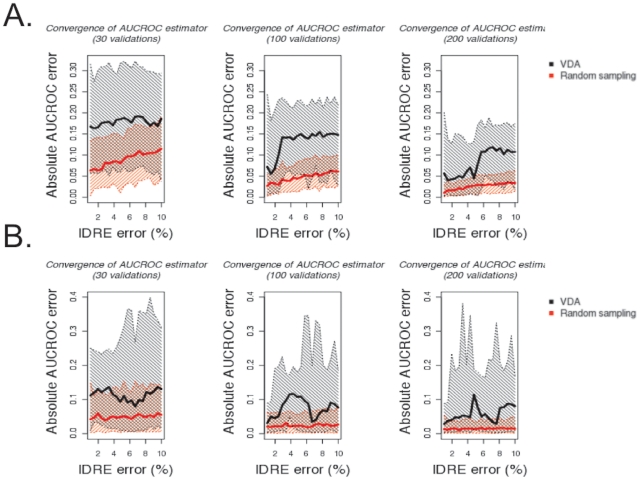
Distributions of maximum absolute error in inferring the AUCROC performance of IDRE algorithms for a fixed number of validations. A-B. The current implementation of VDA leads to larger absolute errors in the estimation of the AUCROC performance. This is due to the design of the current implementation that tries to enforce differences between algorithms. The statistics from the VDA validation set are shown in black and the statistics from the randomly sampled validation set are shown in red. The median absolute AUCROC error for each IDRE error probability is shown as a bold line. The shaded regions correspond to the 95% confidence interval A. Distribution of maximum absolute differences of AUCROC performances between 5 IDRE algorithms with true AUCROC of 0.7. As expected the median absolute AUCROC error of random sampling increases as the probability of errors in the IDRE increases. The same is true for the VDA, although the AUCROC errors are on average three times larger than the random sampling. The distortions in the VDA trend are probably due to the deterministic nature of the selection procedure. B. Distribution of maximum absolute differences of AUCROC performances between 5 IDRE algorithms with a true AUCROC of 0.7. In this case, we added 5 additional RDFA algorithms (AUCROCs = {0.1, 0.3, 0.5, 0.7, 0.9}) as a confounding effect. The VDA selection exhibits larger distortions due to the smaller number of experiments used to actually discriminate between IDRE algorithms, since some of the selected experiments are now used to discriminate between the additional RDFAs algorithms.

We demonstrate the discriminatory power of VDA approaches for independent RDFA algorithms. Another desirable property of validation sets is the ability to detect whether two algorithms have identical performances, thus letting the researcher chooses freely without loss of quality. To compare the ability of a VDA approach in detecting converging performances, we simulate predictions from IDRE algorithms (Identical Detectors with Random Errors, see Methods for definition) with identical performances and compare the distribution of differences between the true and estimated AUCROC to the error obtained from random sampling. As expected, in this constructed dataset where all algorithms have the same performance, the VDA set has wider error distributions than random sampling and slower convergence (Figure 2A). The same result holds true for the case in which only a fraction of algorithms have identical performances (Figure 2B). This is a direct consequence of our GVDA algorithm implementation, such that the validation set is chosen to maximize differences between algorithms. Remarkably, the median error from random sampling is always within the 95% confidence interval of the error from the VDA validation set.

### Implementation of VDA to discriminate between gene expression profiles of melanoma samples and profiles of other malignancies

The RDFA and IDRE algorithms are extremely useful for studying convergence of validation set selection algorithms. However, these are classes of methods with limited practical use. To demonstrate a practical application VDA in a common experimental setting we compare gene expression profiles of one tumor type to other tumor types. Genes with high expression levels in tumors are often considered candidate targets for novel drugs [20]–[23]. We assume that a dataset of gene expression profiles of tumor samples has been affected by mislabeling of the cancer class. Prior to repeating all experiments, it may be a good idea to verify if a machine-learning tool can recover the missing label. This can be done by training selected machine-learning tools on a set of correctly labeled data, inferring the cancer class for the mislabeled samples and designing a few experiments, i.e. validating the tumor type by immuno-histochemistry on the remaining part of the sample, or on the accompanying tissue to establish the organ of origin, instead of repeating the entire study, in order to validate the predictions. Repetition of all the experiments can be very expensive, therefore it is desirable to minimize the number of required validations.

**Figure 3:**
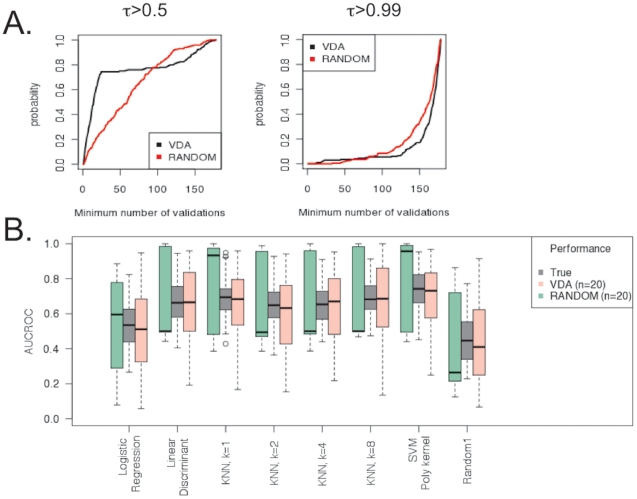
Comparison of performance inference of VDA and random sampling (RANDOM) strategies on experimental data. A-B. VDA and random sampling (RANDOM) were used to select predictions to validate the ability of seven algorithms, plus a random classifier (Random1), to infer melanoma status (versus non melanoma) from gene expression profiling data (see Results). A. Probability distribution of achieving a target τ-statistics between the inferred ranking of performances and the true performances with at most a fixed maximum number of validation experiments. VDA (in black) is more powerful than random sampling (in red) at enabling a τ = 0.5, or better. In particular a VDA validation set can achieve τ>0.5 with less than 30 experiments in more than 70% of the tests, while random sampling may result in twice as many validations to achieve the same performance (left panel). However, to obtain higher correlations (τ>0.99), additional experiments are needed, and VDA would require, on average, 10 more validations (right panel). B. Boxplots of algorithmic performances for each of the algorithms used in the tests across 500 tests. With only 20 validations, VDA (pink) shows estimated performance distributions that are very close to the actual AUCROC distributions of the algorithms (grey). As expected, random sampling (RANDOM, green) exhibits higher variance in the AUCROC estimation than VDA (pink) at 20 validations. Interestingly, SVM with a polynomial kernel was on average better than the other algorithms.

We use 20 randomly selected predictions to train seven state-of-the-art machine-learning algorithms to predict whether the cancer class is melanoma. In contrast to other tumors, primary melanoma lesions can be detected early, when the tumor is very small and thus very little material may be available for additional high-throughput analysis. We then collect the predictions for the remaining 178 cases and determine whether the use of a VDA approach is beneficial in terms of cost, relative to a random sampling strategy to select predictions for validation. We conduct this simulation 500 times, each time using a different training set of 20 predictions selected at random. Since KNN and SVM are affected by the dimensionality of the data, we reduce the set of genes to a pool of 100 genes, selected at random for every simulation. Despite this arbitrary choice, the top algorithms held good performances, suggesting that tumors from different organs exhibit global differences in their gene expression profiles (Figure 3B).

Using our GVDA algorithm we achieve a good correlation with the true ranking of the algorithms (τ = 0.5, Kendall τ statistics) faster than using a random selection strategy in at least 80% of the tests (Figure 3A, left). This is also reflected in the high agreement between performance estimates obtained after 20 validations and the performance obtained by validating all instances (see section D of Appendix S1). The VDA set exhibits smaller variances and estimates that are closer to the true performances (Figure 3B). However, some algorithms have similar performances (Figure 3B), leading to a slower convergence of the VDA approach to the exact ranking (Figure 3A, right). Importantly, the difference in number of additional experiments required by the randomly selected validation set to achieve a good correlation (τ = 0.5) is larger than the additional experiments required by the VDA validation set to achieve perfect ranking. Also, with as little as 20 validation experiments, the VDA validation set can already give an indication that SVM is probably the best algorithm for the task.

## Discussion

The present study shows how sampling strategies other than random sampling can yield better results in the context of evaluating machine learning applications to biological and medical fields. The novelty and strength of this alternative sampling strategy are in the design of validation sets that maximize the difference in predictions between algorithms of interest. In contrast to other performance assessment techniques, such as cross-validation, the VDA procedure is intended to serve as a guide in the design of independent validation datasets to test the performance of existing algorithms. Using the validations from the VDA dataset to fine-tune internal parameters of any algorithm is strongly discouraged, as it may lead to biases in the application of Equation 5 as well as overfitting estimates of accuracy.

The VDA procedure borrows principles from importance sampling in Monte Carlo simulations [24] and from active learning [25], [26]. Similar to importance sampling, a more efficient sampling technique replaces the original mechanism; this achieves quicker variance reduction in the estimation of the desired quantity. This change of sampling technique requires that the function for estimating the desired quantity is modified accordingly. In this sense, we reformulate the AUCROC estimator (Equation 5) to reflect the fact that the VDA sampling strategy explores different partitions of the data according to their ability to discriminate between algorithms. In active learning, the ground truth of a set of predictions is demanded from the oracle in order to improve a classifier or a learning task. Similar to the predictions in the VDA validation set, these predictions have the expectation of leading to maximum performance gain, such as increasing the discriminatory power (see section D of Appendix S1). However, VDA is generally not intended to be an online or dynamic procedure, nor is its selected validation set supposed to be used to optimize any parameters.

Recent developments in machine learning have suggested that the use of combinations of suboptimal algorithms, or weak learners, may result in a super-algorithm with improved performance [27], [28]. The possibility to build such classifiers is not in contrast to the basic idea of using VDA. As there is a combinatorially large number of ways to combine algorithms together, VDA should still be employed to assess and compare the performances of the super-algorithms of interest, while carefully avoiding the use of VDA validation dataset to build such super-algorithms, which would overfit the super-algorithms to the validation data.

In summary, the main advantage of VDA relative to random sampling is that VDA constructs a partition set of the predictions based on global comparisons between algorithms. In many practical applications such a partition will contain largely unbalanced subsets. VDA-based sampling from these subsets enables quicker evaluation of different algorithmic configurations. In addition, VDA ignores uninformative subsets, or subsets that are too small to determine a significant change in the performance estimate, thus effectively reducing the number of samples needed to provide reliable ranking of performances.

## Methods

### Datasets

In the present study we use a publicly available dataset of tumor gene expression that is frequently used to test machine learning applications [29]. The dataset comprises 16,063 genes and 198 instances, corresponding to 198 tumor samples. The instances are divided into 14 cancer classes. We use this dataset as an illustrative example. The algorithms' task is to predict whether the tumor sample was melanoma rather than a different type of cancer.

### Algorithms

In the present study we employ four standard machine-learning methods implemented in the R statistical software (http://www.r-project.org/). These methods are k-nearest-neighbors (function knn in the package class), support vector machines (function ksvm in the package kernlab), logistic regression (function glm), and linear discriminant analysis (function lda in the package MASS of R statistical software). These four methods are used in the illustrative example of predicting whether a tumor sample was melanoma based on its gene expression. In addition we designed two groups of *ad hoc* methods to study convergence rates and discriminatory power of the VDA approach. These groups of *ad hoc* methods are described below.

### Random detectors with fixed AUCROC - RDFA

RDFA(*a*) methods are used to study the speed and robustness of ranking of the algorithmic performances under different sampling strategies. The use of RDFAs simulates the use of independent algorithms. RDFAs are constructed such that their AUCROC is equal to *a*, although the ratio 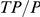 and 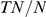 may be different for equal choices of *a*. An RDFA prediction is constructed by selecting a random set *s* of instances from *U'*, with 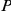 positives and 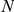 negatives. A random number 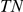 of negatives is assigned the correct class label, such that 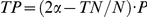 is an integer. Finally, a number 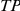 of positives is assigned the correct class label. The advantage of using this class of methods is that the reported results are independent of any property of the measured signal and any other method in the class.

### Identical detectors with random errors – IDRE

IDRE(*p,a,n*) methods correspond to the classifier whose predictions have been corrupted by errors with the probability *p*. The purpose of this class of methods is the study of robustness to noise and convergence rates. We construct each group of *n* IDRE(*p,a,n*) predictors from one RDFA(*a*) realization, switching the predicted label in random fractions *p* of the instances.

### Implementation and availability

GVDA and EVDA have been implemented in Java and are freely available at http://sourceforge.net/projects/klugerlab/files/VDA/

## Supporting Information

Appendix S1 **Technical appendix.** A technical appendix consisting of four sections: A) The validation problem, B) Choice of validation set using the greedy VDA algorithm, C) Estimation of the AUCROC for a VDA set, and D) Recommended sizes for VDA validation sets.
(PDF)
