# Supplementary material for: VDA, a Method of Choosing a Better Algorithm with Fewer Validations
Source: PLoS One. 2011 Oct 12;6(10):e26074. doi: 10.1371/journal.pone.0026074 (PMC3192143; doi:10.1371/journal.pone.0026074)
Supplement: Appendix S1 — Technical appendix. A technical appendix consisting of four sections: A) The validation problem, B) Choice of validation set using the greedy VDA algorithm, C) Estimation of the AUCROC for a VDA set, and D) Recommended sizes for VDA validation sets. (PDF) [file pone.0026074.s001.pdf]

Technical appendix to: VDA, a method of choosing a better algorithm with fewer validations

Francesco Strino[1,†], Fabio Parisi[1,†] and Yuval Kluger[1*]

[1]Department of Pathology, Yale University School of Medicine, 333 Cedar St., New Haven, CT 06520, USA

Appendix A – The validation problem

A dataset is an ensemble of instances that can be analyzed to determine the presence or absence of events, which are the manifestations of an underlying phenomenon investigated in an experiment. This analysis can be performed assuming that patterns in the dataset are associated with the events of the phenomenon. Hence, algorithms can be designed to analyze the data and infer, for each instance, whether an event has occurred. A set of M algorithms designed to analyze the data will therefore generate a set of M predictions for each instance in the dataset. The process of assessing algorithmic performance using experimental procedures that are independent from the procedures used to generate the dataset is termed validation procedure, or validation. Each experiment that verifies the goodness of predictions for a single instance is termed validation experiment, and the collection of instances that are verified is the validation set. In many practical settings, due to operative costs, validation is performed only once on a set of instances often containing several orders of magnitude fewer instances than the entire dataset.

Appendix B – choice of validation set using the greedy VDA algorithm

We consider this toy example in which there are M=4 algorithms (A_1,..., A_4) and 10 instances in the dataset U. The table below represents the predictions of these algorithms:

instance	A_1	A_2	A_3	A_4
u_1	1	1	0	0
u_2	1	1	1	0
u_3	0	0	0	1
u_4	1	1	0	0
u_5	1	0	0	1
u_6	1	0	0	1
u_7	0	0	1	1
u_8	0	0	0	1
u_9	1	1	0	0
u_{10}	0	0	1	1

Instances where the predictions from all algorithms are the same are grouped together and the resulting clusters are used to partition the dataset. The values of $\delta_{ij}(t)$ (an indicator variable that takes the value 1 if the algorithms i and j have a discordant prediction for instance t, and 0 otherwise) are also shown.

partition	instances	A_1	A_2	A_3	A_4	Cardinality	$\delta_{ij}(t)$
k_1	u_1, u_4, u_9	1	1	0	0	3	$\begin{array}{\|c\|c\|c\|c\|}\hline 0 & 0 & 1 & 1 \\ \hline 0 & 0 & 1 & 1 \\ \hline 1 & 1 & 0 & 0 \\ \hline 1 & 1 & 0 & 0 \\ \hline \end{array}$
k_2	u_2	1	1	1	0	1	$\begin{array}{\|c\|c\|c\|c\|}\hline 0 & 0 & 0 & 1 \\ \hline 0 & 0 & 0 & 1 \\ \hline 0 & 0 & 0 & 1 \\ \hline 1 & 1 & 1 & 0 \\ \hline \end{array}$
k_3	u_3, u_8	0	0	0	1	2	$\begin{array}{\|c\|c\|c\|c\|}\hline 0 & 0 & 0 & 1 \\ \hline 0 & 0 & 0 & 1 \\ \hline 0 & 0 & 0 & 1 \\ \hline 1 & 1 & 1 & 0 \\ \hline \end{array}$
k_4	u_5, u_6	1	0	0	1	2	$\begin{array}{\|c\|c\|c\|c\|}\hline 0 & 1 & 1 & 0 \\ \hline 1 & 0 & 0 & 1 \\ \hline 1 & 0 & 0 & 1 \\ \hline 0 & 1 & 1 & 0 \\ \hline \end{array}$
k_5	u_7, u_{10}	0	0	1	1	2	$\begin{array}{\|c\|c\|c\|c\|}\hline 0 & 0 & 1 & 1 \\ \hline 0 & 0 & 1 & 1 \\ \hline 1 & 1 & 0 & 0 \\ \hline 1 & 1 & 0 & 0 \\ \hline \end{array}$

In this example we want to demonstrate how to select a validation set of size 3 using the greedy VDA (GVDA) algorithm.

In the first step, the validation dataset V_0 is empty, the set of instances Z_0 is the whole dataset U and the Hamming distance matrix $H_{ij}(V_0)$ is zero everywhere:

$$V_0=\{\} \qquad Z_0=\{u_1, u_2, u_3, u_4, u_5, u_6, u_7, u_8, u_9, u_{10}\} \qquad H_{ij}(V_0)= \begin{array}{|c|c|c|c|}\hline 0 & 0 & 0 & 0 \\ \hline 0 & 0 & 0 & 0 \\ \hline 0 & 0 & 0 & 0 \\ \hline 0 & 0 & 0 & 0 \\ \hline \end{array}$$

For each instance, we evaluate the function $\sum_{i=1}^{M}\sum_{j=1}^{M}\left(\frac{1}{M^2}\right)^{\delta_{ij}(t)+H_{ij}(V_0)}$ (see Equation 4), which depends on the values of $H_{ij}(V_0)+\delta_{ij}(t)$ (shown in bold when $\delta_{ij}(t)=1$, i.e.

when the addition of instance t increases the Hamming distance between algorithm i and j). GVDA selects the instance to be included in the validation set from the set of instances that minimize the function $\sum_{i=1}^{M}\sum_{j=1}^{M}\left(\frac{1}{4^2}\right)^{\delta_{ij}(t)+H_{ij}(V_0)}$. In this case, the partitions k_1, k_4 and k_5 (in gray) minimize the target function, so an instance from these partitions is chosen at random and inserted in the validation set

partition	instances	A_1	A_2	A_3	A_4	$\delta_{ij}(t)+H_{ij}(V_0)$	$\sum_{i=1}^{M}\sum_{j=1}^{M}\left(\frac{1}{4^2}\right)^{\delta_{ij}(t)+H_{ij}(V_0)}$
k_1	u_1, $\mathbf{u_4}$, u_9	1	1	0	0	$\begin{bmatrix} 0 & 0 & \mathbf{1} & \mathbf{1} \\ 0 & 0 & \mathbf{1} & \mathbf{1} \\ \mathbf{1} & \mathbf{1} & 0 & 0 \\ \mathbf{1} & \mathbf{1} & 0 & 0 \end{bmatrix}$	17/2 = 8.5
k_2	u_2	1	1	1	0	$\begin{bmatrix} 0 & 0 & 0 & \mathbf{1} \\ 0 & 0 & 0 & \mathbf{1} \\ 0 & 0 & 0 & \mathbf{1} \\ \mathbf{1} & \mathbf{1} & \mathbf{1} & 0 \end{bmatrix}$	83/8 = 10.375
k_3	u_3, u_8	0	0	0	1	$\begin{bmatrix} 0 & 0 & 0 & \mathbf{1} \\ 0 & 0 & 0 & \mathbf{1} \\ 0 & 0 & 0 & \mathbf{1} \\ \mathbf{1} & \mathbf{1} & \mathbf{1} & 0 \end{bmatrix}$	83/8 = 10.375
k_4	u_5, u_6	1	0	0	1	$\begin{bmatrix} 0 & \mathbf{1} & \mathbf{1} & 0 \\ \mathbf{1} & 0 & 0 & \mathbf{1} \\ \mathbf{1} & 0 & 0 & \mathbf{1} \\ 0 & \mathbf{1} & \mathbf{1} & 0 \end{bmatrix}$	17/2 = 8.5
k_5	u_7, u_{10}	0	0	1	1	$\begin{bmatrix} 0 & 0 & \mathbf{1} & \mathbf{1} \\ 0 & 0 & \mathbf{1} & \mathbf{1} \\ \mathbf{1} & \mathbf{1} & 0 & 0 \\ \mathbf{1} & \mathbf{1} & 0 & 0 \end{bmatrix}$	17/2 = 8.5

In this example, $\mathbf{u_4}$ (in bold) was selected and the sets V and Z are updated.

$V_1=\{u_4\}$ $\quad$ $Z_1=\{u_1, u_2, u_3, u_5, u_6, u_7, u_8, u_9, u_{10}\}$ $\quad$ $H_{ij}(V_1)=\begin{bmatrix} 0 & 0 & 1 & 1 \\ 0 & 0 & 1 & 1 \\ 1 & 1 & 0 & 0 \\ 1 & 1 & 0 & 0 \end{bmatrix}$

We now repeat the evaluation step in order to find the second instance to be included in the validation set:

partition	instances	A_1	A_2	A_3	A_4	$\delta_{ij}(t) + H_{ij}(V_1)$	$\sum_{i=1}^{M}\sum_{j=1}^{M}\left(\frac{1}{4^2}\right)^{\delta_{ij}(t)+H_{ij}(V_1)}$
k_1	u_1, u_9	1	1	0	0	$\begin{bmatrix} 0 & 0 & \mathbf{2} & \mathbf{2} \\ 0 & 0 & \mathbf{2} & \mathbf{2} \\ \mathbf{2} & \mathbf{2} & 0 & 0 \\ \mathbf{2} & \mathbf{2} & 0 & 0 \end{bmatrix}$	257/32 = 8.0313
k_2	u_2	1	1	1	0	$\begin{bmatrix} 0 & 0 & 1 & \mathbf{2} \\ 0 & 0 & 1 & \mathbf{2} \\ 1 & 1 & 0 & \mathbf{1} \\ \mathbf{2} & \mathbf{2} & \mathbf{1} & 0 \end{bmatrix}$	409/64 ≈ 6.3906
k_3	u_3, u_8	0	0	0	1	$\begin{bmatrix} 0 & 0 & 1 & \mathbf{2} \\ 0 & 0 & 1 & \mathbf{2} \\ 1 & 1 & 0 & \mathbf{1} \\ \mathbf{2} & \mathbf{2} & \mathbf{1} & 0 \end{bmatrix}$	409/64 ≈ 6.3906
k_4	u_5, $\mathbf{u_6}$	1	0	0	1	$\begin{bmatrix} 0 & \mathbf{1} & \mathbf{2} & 1 \\ \mathbf{1} & 0 & 1 & \mathbf{2} \\ \mathbf{2} & 1 & 0 & \mathbf{1} \\ 1 & \mathbf{2} & \mathbf{1} & 0 \end{bmatrix}$	289/64 ≈ 4.5156
k_5	u_7, u_{10}	0	0	1	1	$\begin{bmatrix} 0 & 0 & \mathbf{2} & \mathbf{2} \\ 0 & 0 & \mathbf{2} & \mathbf{2} \\ \mathbf{2} & \mathbf{2} & 0 & 0 \\ \mathbf{2} & \mathbf{2} & 0 & 0 \end{bmatrix}$	257/32 ≈ 8.0313

In this step, the instances from partitions k_4 minimize the target function and $\mathbf{u_6}$ was selected. The set *V* and *Z* are updated:

$V_2=\{u_4, u_6\}$ $\quad$ $Z_2=\{u_1, u_2, u_3, u_5, u_7, u_8, u_9, u_{10}\}$ $\quad$ $H_{ij}(V_2)= \begin{bmatrix} 0 & 1 & 2 & 1 \\ 1 & 0 & 1 & 2 \\ 2 & 1 & 0 & 1 \\ 1 & 2 & 1 & 0 \end{bmatrix}$

The evaluation step is now repeated a third time in order to find the last instance to be included in the validation set:

partition	instances	A_1	A_2	A_3	A_4	$\delta_{ij}(t) + H_{ij}(V_2)$	$\sum_{i=1}^{M}\sum_{j=1}^{M}\left(\frac{1}{4^2}\right)^{\delta_{ij}(t)+H_{ij}(V_2)}$
k_1	u_1, u_9	1	1	0	0	$\begin{array}{cccc} 0 & 1 & \mathbf{3} & \mathbf{2} \\ 1 & 0 & \mathbf{2} & \mathbf{3} \\ \mathbf{3} & \mathbf{2} & 0 & 1 \\ \mathbf{2} & \mathbf{3} & 1 & 0 \end{array}$	4369/1024 ≈ 4.2666
k_2	u_2	1	1	1	0	$\begin{array}{cccc} 0 & 1 & 2 & \mathbf{2} \\ 1 & 0 & 1 & \mathbf{3} \\ 2 & 1 & 0 & \mathbf{2} \\ \mathbf{2} & \mathbf{3} & \mathbf{2} & 0 \end{array}$	8753/2048 ≈ 4.2739
k_3	u_3, u_8	0	0	0	1	$\begin{array}{cccc} 0 & 1 & 2 & \mathbf{2} \\ 1 & 0 & 1 & \mathbf{3} \\ 2 & 1 & 0 & \mathbf{2} \\ \mathbf{2} & \mathbf{3} & \mathbf{2} & 0 \end{array}$	8753/2048 ≈ 4.2739
k_4	u_5	1	0	0	1	$\begin{array}{cccc} 0 & \mathbf{2} & \mathbf{3} & 1 \\ \mathbf{2} & 0 & 1 & \mathbf{3} \\ \mathbf{3} & 1 & 0 & \mathbf{2} \\ 1 & \mathbf{3} & \mathbf{2} & 0 \end{array}$	4369/1024 ≈ 4.2666
k_5	$\mathbf{u_7}$, u_{10}	0	0	1	1	$\begin{array}{cccc} 0 & 1 & \mathbf{3} & \mathbf{2} \\ 1 & 0 & \mathbf{2} & \mathbf{3} \\ \mathbf{3} & \mathbf{2} & 0 & 1 \\ \mathbf{2} & \mathbf{3} & 1 & 0 \end{array}$	4369/1024 ≈ 4.2666

In the third step, the instances from partitions k_1, k_4 and k_5 minimize the target function so an instance from these partitions is chosen at random and inserted in the validation set. In this example, $\mathbf{u_7}$ was selected and the desired validation set is $V_3 = \{u_4, u_6, u_7\}$.

Appendix C - Estimation of the AUCROC for a VDA set

Validation Discriminant Analysis is a non-random sampling procedure to select instances for experimental validation. The inherent selection bias introduced by VDA requires a correction to enable accurate estimation of algorithmic performances. Given a partition set of the data, namely a group of non-overlapping and non-empty sets of instances, such that the union of these sets corresponds to the entire data, the AUCROC in Equation 1 can be rewritten as the AUCROC of weighted averages of *TP* and *TN* over the partition set of the data. For a given algorithm:

$$p = \frac{1}{2}\left(\sum_{k \in W}\left(\frac{P_k}{P}\cdot\frac{TP_k}{P_k}\right) + \sum_{k \in W}\left(\frac{N_k}{N}\cdot\frac{TN_k}{N_k}\right)\right), \qquad \text{(S1)}$$

with W, the partition set of U, P_k and N_k the number of predicted positives and predicted negatives in the k-th partition, and TP_k and TN_k the number of true positives and true negatives in the k-th partition of U. Typically, in a validation experiment, only a subset of instances from each partition is tested.

A natural partition method in the VDA approach is to divide the dataset into clusters, which share the same fingerprint (see example below). Specifically, a fingerprint is an M-dimensional binary vector whose i-th element indicates whether the prediction made by the i-th algorithm for each of these instances is classified as positive (1) or negative (0). However, the equations derived in this work are valid for any partition strategy. Under the assumption of unbiased sampling within each partition, the AUCROC can be calculated from estimates of *TP*, *TN*, *P* and *N* derived using the law of total probability over the partition set W. Importantly, we define

the set $W' \subset W$ as the subset of W for which at least one validation experiment is available. We can thus write the estimates for *TP*, *TN*, *P* and *N* as follows:

$$\begin{aligned}
\overline{TP} &= R \cdot \sum_{k} p\left(TP'_k \middle| P'_k, W_k\right) p\left(P'_k \middle| W_k\right) p\left(W_k\right) \cong R \cdot \sum_{k \in W'} \left(\frac{TP'_k}{P'_k} \cdot \frac{P'_k}{P'_k + N'_k} \cdot \frac{C_k}{\sum_{j \in W'} C_j} \right) \\
\overline{TN} &= R \cdot \sum_{k} p\left(TN'_k \middle| N'_k, W_k\right) p\left(N'_k \middle| W_k\right) p\left(W_k\right) \cong R \cdot \sum_{k \in W'} \left(\frac{TN'_k}{N'_k} \cdot \frac{N'_k}{P'_k + N'_k} \cdot \frac{C_k}{\sum_{j \in W'} C_j} \right) \\
\overline{P} &= R \cdot \sum_{k} p\left(P'_k \middle| W_k\right) p\left(W_k\right) \cong R \cdot \sum_{k \in W'} \left(\frac{P'_k}{P'_k + N'_k} \cdot \frac{C_k}{\sum_{j \in W'} C_j} \right) \\
\overline{N} &= R \cdot \sum_{k} p\left(N'_k \middle| W_k\right) p\left(W_k\right) \cong R \cdot \sum_{k \in W'} \left(\frac{N'_k}{P'_k + N'_k} \cdot \frac{C_k}{\sum_{j \in W'} C_j} \right)
\end{aligned} \quad , \quad \text{(S2)}$$

where R is the total number of detectable instances in the data, i.e. the cardinality of U; W' is the subset of W for which at least one validation experiment is available; C_k is the cardinality of the k-th partition W_k, namely the number of instances in the partition; P'_k and N'_k are the experimentally validated positives and negatives in the k-th partition; TP'_k and TN'_k are the confirmed true positives and true negatives in the k-th partition. These estimates lead to the following general formulation of the AUCROC estimate for any partition set *W*:

$$p = \frac{1}{2} \left(\frac{\sum_{k \in W'} \left(\frac{C_k}{\sum_{j \in W'} C_j} \cdot \frac{P'_k}{P'_k + N'_k} \cdot \frac{TP'_k}{P'_k} \right)}{\sum_{k \in W'} \left(\frac{C_k}{\sum_{j \in W'} C_j} \cdot \frac{P'_k}{P'_k + N'_k} \right)} + \frac{\sum_{k \in W'} \left(\frac{C_k}{\sum_{j \in W'} C_j} \cdot \frac{N'_k}{P'_k + N'_k} \cdot \frac{TN'_k}{N'_k} \right)}{\sum_{k \in W'} \left(\frac{C_k}{\sum_{j \in W'} C_j} \cdot \frac{N'_k}{P'_k + N'_k} \right)} \right) \quad . \quad \text{(S3)}$$

Using Equation S3 we can calculate the performance of each algorithm from the VDA set. Although Equation S3 can be used with any partition set, the GVDA and EVDA algorithms are based on a partition set W' that divides the data into clusters where all the instances within a given cluster share the same fingerprint.

The motivation for using Equation S3 to assess algorithmic performance stems from the fact that algorithms remap the space of the data to a new space of predictions where different underlying probabilities of event occurrence are encoded in the structure of the remapped data. In addition, most algorithms are not random, but intelligently designed to capture at least some part of the data structure in order to make predictions. Altogether, combinations of predictions from different algorithms typically reflect structures in the data, and thus have hidden relationship to the unknown underlying probability of an event. However, we note that when all the algorithms are random classifiers, there is no advantage in using formula S3 because sampling with the VDA approach leads to equivalent results as random sampling. The true novelty of Equation S3 is the use of a partitioning scheme in order to enable grouping instances into sets of predictions with similar underlying probability of events. Leveraging on the law of total probabilities, this partitioning scheme allows a robust estimate of the expected total number of TP and TN. Moreover, increasing the number of algorithms in the analysis improves the robustness of the performance estimation based on the partitioning scheme as described in Equation S3. To improve robustness of performance estimates using GVDA (or EVDA), we weight each partition according to its frequency, such that in

case of equality of scores in Equation S3 we sample preferentially from larger partitions.

In principle, D, the set of all instances for which at least two algorithms give discordant predictions, should be sufficient to discriminate between algorithms. In general, however, the choice of sampling from D may introduce an unknown bias in the AUCROC estimation when compared to the estimation from random samples in U. To avoid this issue, two *ad hoc* algorithms are added by default to the set of algorithms: one algorithm assumes that every prediction is a positive (AllTrue), and the other assumes that every prediction is a negative (AllFalse). AllTrue has sensitivity equal to 1 and specificity equal to 0. AllFalse has sensitivity equal to 0 and specificity equal to 1. These two *ad hoc* algorithms have AUCROCs corresponding to the expected AUCROC of a random classifier (p=0.5), corresponding to the average of specificity and sensitivity. By adding these two algorithms, the set D will comprise all instances of U. Furthermore, when AllFalse is included in the set of algorithms, the number of instances classified as positives by each algorithm is ensured to be equal or greater than h_m (the minimum Hamming distance between all pair of algorithms in the VDA validation set). Likewise, the presence of AllTrue ensures that at least h_m instances in the VDA set were classified as negatives.

In the most general case, the error of the AUCROC estimates resulting from the exclusion of some partitions is unknown and it is at most equal to 1/2, corresponding to the extreme and unlikely case when the validation experiments

consist only of a collection of positives or of negatives, but not both. This observation holds for both VDA and random sampling approaches.

An illustrative example of calculating the AUCROC at the operative point

We consider an example where the dataset is made of 100 instances out of which 20 are validated. We partition the data into fingerprints, each representing a prediction profile of all algorithms (e.g., in partition k_1 the prediction of the four algorithms is given by the fingerprint A_1=1,A_2=1, A_3=0 and A_4=0).

partition	cardinality	A_1	A_2	A_3	A_4	Positive validations	Negative validations	Validated instances
k_1	45	1	1	0	0	4	1	5
k_2	10	1	1	1	0	1	1	2
k_3	5	0	0	0	0	0	0	0
k_4	25	1	0	0	1	1	7	8
k_5	15	0	0	1	1	1	4	5
total	100					7	13	20

The corrected numbers of positives (P), negatives (N), true positives (TP) and true negatives (TN) are estimated using Equation 5:

$$\overline{P} = 100 \cdot \sum_{k \in \{k_1,k_3,k_4,k_5\}} \left(\frac{P'_k}{P'_k + N'_k} \cdot \frac{C_k}{\sum_{j \in \{k_1,k_3,k_4,k\}} C_j} \right) = 100 \cdot \left(\frac{4}{5} \cdot \frac{45}{45+10+25+15} + \frac{1}{2} \cdot \frac{10}{95} + \frac{1}{8} \cdot \frac{25}{95} + \frac{1}{5} \cdot \frac{15}{95} \right) = \frac{1885}{38} \cong 49.6$$

$$\overline{N} = 100 \cdot \sum_{k \in \{k_1,k_3,k_4,k_5\}} \left(\frac{N'_k}{P'_k + N'_k} \cdot \frac{C_k}{\sum_{j \in \{k_1,k_3,k_4,k\}} C_j} \right) = 100 \cdot \left(\frac{1}{5} \cdot \frac{45}{95} + \frac{1}{2} \cdot \frac{10}{95} + \frac{7}{8} \cdot \frac{25}{95} + \frac{4}{5} \cdot \frac{15}{95} \right) = \frac{1915}{38} \cong 50.4$$

For algorithm 1, the number of true positives and negatives are estimated using Equation 5:

$$\overline{TP_1} = 100 \cdot \sum_{k\in W'} \left(\frac{TP'_k}{P'_k + N'_k} \cdot \frac{C_k}{\sum_{j\in W'} C_j} \right) = 100 \cdot \left(\frac{4}{5} \cdot \frac{45}{95} + \frac{1}{2} \cdot \frac{10}{95} + \frac{1}{8} \cdot \frac{25}{95} + \frac{0}{5} \cdot \frac{15}{95} \right) = \frac{1756}{38} \approx 46.5$$

$$\overline{TN_1} = 100 \cdot \sum_{k\in W'} \left(\frac{TN'_k}{P'_k + N'_k} \cdot \frac{C_k}{\sum_{j\in W'} C_j} \right) = 100 \cdot \left(\frac{0}{5} \cdot \frac{45}{95} + \frac{0}{2} \cdot \frac{10}{95} + \frac{0}{8} \cdot \frac{25}{95} + \frac{4}{5} \cdot \frac{15}{95} \right) = \frac{240}{19} \approx 12.6$$

The AUCROC at the operative point for algorithm 1 can then be calculated as:

$$p_1 = \frac{1}{2}\left(\frac{\overline{TP_1}}{\overline{P}} + \frac{\overline{TN_1}}{\overline{N}} \right) \cong \frac{1}{2}\left(\frac{46.5}{49.6} + \frac{12.6}{50.4} \right) = \frac{1}{2}(0.94 + 0.25) = 0.59$$

Analogously, for algorithm 2:

$$\overline{TP_2} = 100 \cdot \sum_{k\in W'} \left(\frac{TP'_k}{P'_k + N'_k} \cdot \frac{C_k}{\sum_{j\in W'} C_j} \right) = 100 \cdot \left(\frac{4}{5} \cdot \frac{45}{95} + \frac{1}{2} \cdot \frac{10}{95} + \frac{0}{8} \cdot \frac{25}{95} + \frac{0}{5} \cdot \frac{15}{95} \right) = \frac{820}{19} \cong 43.2$$

$$\overline{TN_2} = 100 \cdot \sum_{k\in W'} \left(\frac{TN'_k}{P'_k + N'_k} \cdot \frac{C_k}{\sum_{j\in W'} C_j} \right) = 100 \cdot \left(\frac{0}{5} \cdot \frac{45}{95} + \frac{0}{2} \cdot \frac{10}{95} + \frac{7}{8} \cdot \frac{25}{95} + \frac{4}{5} \cdot \frac{15}{95} \right) = \frac{1355}{38} \cong 35.6$$

$$p_2 = \frac{1}{2}\left(\frac{\overline{TP_2}}{\overline{P}} + \frac{\overline{TN_2}}{\overline{N}} \right) \cong \frac{1}{2}\left(\frac{43.2}{49.6} + \frac{35.6}{50.4} \right) = \frac{1}{2}(0.87 + 0.71) = 0.79$$

Similarly, the performances for algorithm 3 and 4 can be estimated, giving $p_3 \cong 0.41$ and $p_4 \cong 0.21$.

Appendix D - Recommended sizes for VDA validation sets

The required sample size needed to safely verify that a certain algorithm performs better than random is a critical issue. For simplicity we consider two algorithms and a validation set made of an equal number of positives P and negatives N. Under these conditions, the AUCROC of the algorithms (Equation 1) equals their accuracy. We also assume that one of the algorithms is a random classifier with equal probability of predicting positive or negative predictions, thus having an accuracy of 0.5. In this case, discriminating the two algorithms is equivalent to performing a binomial test on the accuracy of the non-random classifier with the null-hypothesis being that the accuracy is equal to 0.5. We thus evaluate the relationship between power of a binomial test, accuracy and sample size. In particular, testing whether the accuracy of the non-random classifier was significantly above 0.5, we find that 30 validated predictions are sufficient to achieve at least 75% power in significantly detecting an algorithm with an accuracy of 0.7; for highly accurate algorithms, e.g. with an accuracy >0.9, only 10 experimental validations are needed to achieve at least 90% power (Figure S1).

Although these results may depend on the specific selection of predictions for validation, and other statistical approaches may hold higher power than a binomial test, they are independent from the selection strategy and are equally valid for both random sampling and VDA within the limitations of the stated assumption of an equal number of positives P and negatives N.

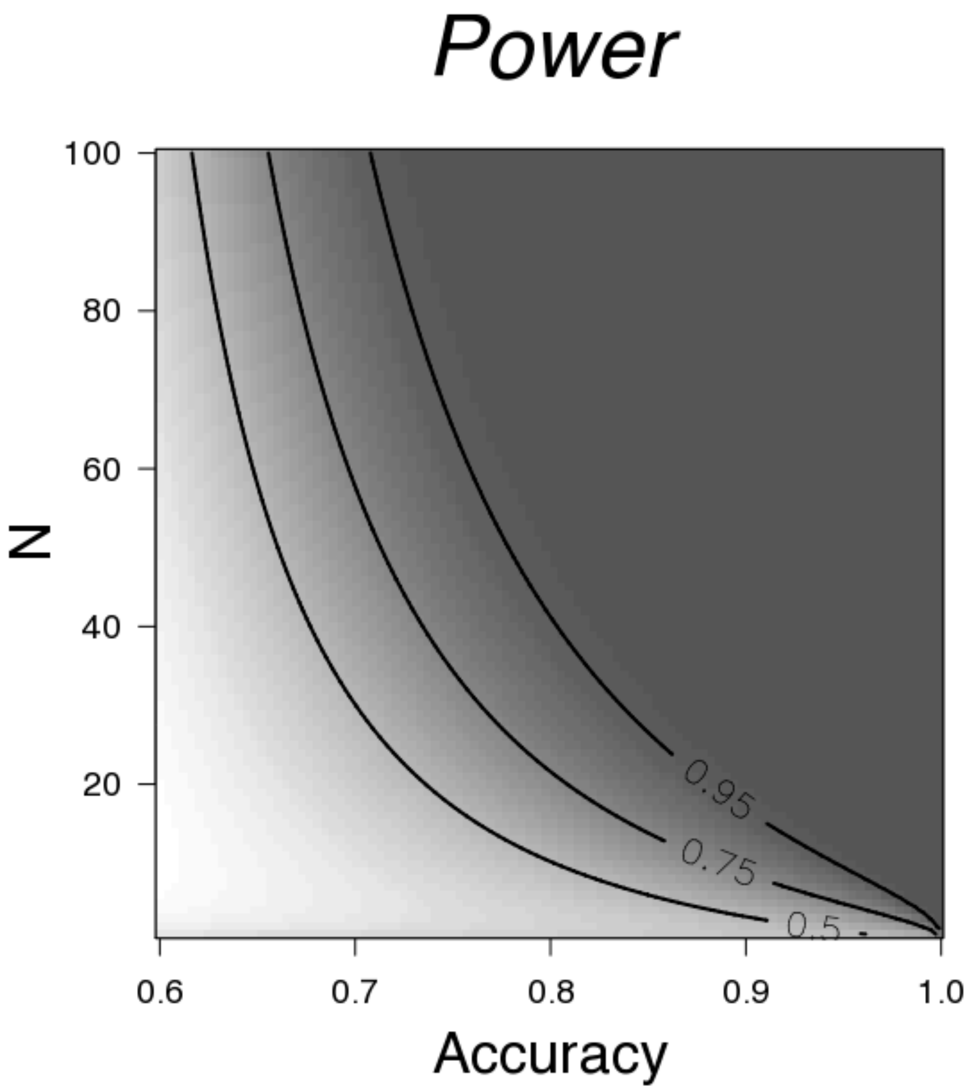

Figure S1 - Discriminatory power of a binomial test as a function of the number of observations (N) and of the true probability of success (Accuracy). The power of the binomial test shown in the figure corresponds to the probability of discriminating a non-random classifier (accuracy shown on the horizontal axis) from a random classifier (accuracy=0.5). To achieve higher powers, larger numbers of experiments are needed. Power is shown in grey scale, with black corresponding to 100% power and white corresponding to 0% power. Isoclines for powers of 95%, 75% and 50% have been added.
